# PCSEL Performance of Type-I InGaAsSb Double-QWs Laser Structure Prepared by MBE

**DOI:** 10.3390/ma12020317

**Published:** 2019-01-21

**Authors:** Hui-Wen Cheng, Shen-Chieh Lin, Zong-Lin Li, Kien-Wen Sun, Chien-Ping Lee

**Affiliations:** 1 Center for Nano Science and Technology, National Chiao Tung University, Hsinchu 300, Taiwan; 2 Department of Electronics Engineering and Institute of Electronics, National Chiao Tung University, Hsinchu 300, Taiwan; z810481@gmail.com (S.-C.L.); martin323261@gmail.com (Z.-L.L.); kwsun@mail.nctu.edu.tw (K.-W.S.); cplee@mail.nctu.edu.tw (C.-P.L.); 3 Department of Applied Chemistry, National Chiao Tung University, Hsinchu 300, Taiwan

**Keywords:** Type-I QW, InGaAsSb/AlGaAsSb, MBE, lattice matched, PCSEL

## Abstract

This paper discusses the issue of controlling the epitaxial growth of mixed group V alloys to form a type-I InGaAsSb/AlGaAsSb double quantum wells (QWs) structure. We also discuss the run-to-run reproducibility of lattice-matched AlGaAsSb alloys and strained In_0.35_Ga_0.65_As_0.095_Sb_0.905_ in terms of growth parameters (V/III ratio, Sb_2_/As_2_ ratio). Molecular beam epitaxy (MBE) was used to grow two type-I InGaAsSb double-QWs laser structures differing only in the composition of the bottom cladding layer: Al_0.85_Ga_0.15_As_0.072_Sb_0.928_ (sample A) and Al_0.5_Ga_0.5_As_0.043_Sb_0.957_ (sample B). Both samples were respectively used in the fabrication of photonic crystal surface-emitting lasers (PCSELs). Sample A presented surface lasing action from circular as well as triangular photonic crystals. Sample B did not present surface lasing due to the deterioration of the active region during the growth of the upper cladding. Our findings underline the importance of temperature in the epitaxial formation of Al_x_Ga_1−x_As_y_Sb_1−y_ in terms of lasing performance.

## 1. Introduction

III-V group semiconductors have attracted considerable attention for their potential applicability in optoelectronic devices. Gallium antimonide (GaSb) and GaSb-related semiconductors have shown the greatest potential for high-performance optoelectronics in the mid-infrared range (2–4 um), due to their high carrier mobility and narrow bandgap. Bandgap energy is a crucial material parameter that is closely linked to the operating wavelength of optoelectronic devices. Gallium arsenide (GaAs) and GaSb both present a zinc blende structure; however, the bandgap of GaSb (0.822 eV at 0 K and 0.725 eV at 300 K) is smaller, giving it superior performance in the mid-infrared range [1].

**Figure 1:**
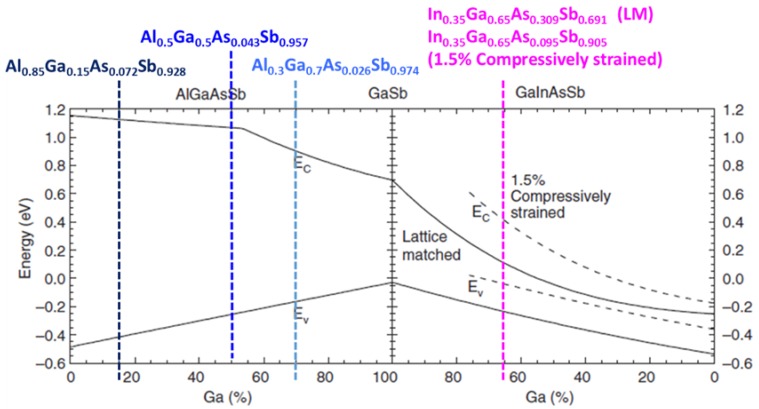
Band-edge positions for AlGaAsSb and InGaAsSb alloys lattice matched to GaSb (solid lines) and 1.5% InGaAsSb alloy under compressive strain (dashed lines). This figure was revised from [5]. The colored dashed lines indicate the target quaternary materials used in this paper.

Multiple quantum well (MQW) structures comprising quaternary materials are important building blocks in modern optoelectronics. Quaternary materials are favored over ternary and binary systems due to their flexibility in achieving the desired strain and fundamental bandgap in the active region of optoelectronic structures (Figure 1) [2]. It is possible that Al_x_Ga_1−x_As_y_Sb_1−y_ and Ga_x_In_1−x_As_y_Sb_l-y_ quaternaries could be used as the basis for optoelectronic devices operating over the wavelength range of two to four um. Quaternary Al_x_Ga_1−x_As_y_Sb_1−y_ alloys are important materials in the fabrication of cladding and waveguides, whereas quaternary Ga_x_In_1−x_As_y_Sb_1−y_ alloys are important constituents in the active region. Furthermore, both alloys can be grown with a lattice matched to commercially available binary GaSb substrates. The lattice constant “a” varies linearly with composition (known as Vegard’s law). Accordingly, the lattice-matching conditions for quaternary alloys on a GaSb substrate can be written as follows [3,4]:

(1) $${\mathrm{Al}}_{x}{\mathrm{Ga}}_{1-x}{\mathrm{As}}_{y}{\mathrm{Sb}}_{1-y}:\;y=\frac{0.0396x}{0.4426+0.0318x}\;(0\leq x\leq 1)$$

(2) $${\mathrm{Ga}}_{x}{\mathrm{In}}_{1-x}{\mathrm{As}}_{y}{\mathrm{Sb}}_{1-y}:\;y=\frac{0.3835-0.3835x}{0.4210+0.0216x}\;(0\leq x\leq 1)$$

Research on injection lasers with an active layer of InGaAsSb began in 1977 [6]. Bell laboratories demonstrated the first room-temperature MBE growth of a InGaAsSb/AlGaAsSb double heterostructures (DH) laser with a wavelength close to 2.2 um [7]. Nonetheless, there remain a number of issues pertaining to the growth of mixed group V alloys (IIIV_1_V_2_ or III_1_III_2_V_1_V_2_ compound semiconductors) via solid-source MBE, including control over the composition and the reproducibility of designed structures, due to a lack of unity in the incorporation of group V beam fluxes. Furthermore, determining the arsenic molar fraction that is to be incorporated depends on the growth temperature, III/V ratio, Sb_2_/As_2_ ratio, and growth rate [8]. The performance of semiconductor devices depends heavily on the structure and optical properties of materials. Thus, much of the research on mixed group V materials has focused on improving crystal quality by minimizing the above-mentioned difficulties. Reducing the growth temperature and using cracker cells for antimony and arsenic has been shown to reduce the concentration of native acceptors and improve the material properties of III-AsSb grown via MBE. Researchers have determined that the optimum temperature range for the growth of high-quality GaSb layers is 500–550 °C [9]. Type-I band structure alignment for DH junctions provides large electron–hole wavefunction overlap (strong radiative recombination), low threshold power, and simplifies the design for QW heterostructure diode lasers [10]. QWs of ~50 to 200 Å allow for the growth of defect-free strained-layer epitaxial (i.e., pseudomorphic) materials with quantum size effects that enable the discretization of energy levels. Thus, we selected Type-I InGaAsSb/AlGaAsSb QW/barrier lasers to produce beams with long wavelengths at room temperature [11].

In this paper, we designed two type-I InGaAsSb/AlGaAsSb QW/barrier laser structures for application in PCSELs. The only difference between the two structures was the composition of the top cladding layer. A photonic crystal (PC) is a periodic optical nanostructure that affects the motion of photons in much the same way that ionic lattices affect electrons in solids. We selected PCSELs for the following reasons: (i) light at the photonic band edge has zero group velocity (band-edge effect in a two-dimensional (2D) PC) and forms a standing wave (2D cavity, due to the 2D distributed feedback laser (DFB) effect, where an internal grating provides distributed feedback for modes whose wavelength satisfies the Bragg condition); (ii) single-mode oscillation over a large area can be obtained as long as gain conditions are met in terms of band-edge resonance; (iii) specific band edges induce in-plane coupling via DFB as well as diffraction in the direction normal to the photonic crystal plane (first-order Bragg diffraction), resulting in surface emission; and (iv) perfect, single longitudinal, and lateral mode oscillation can be achieved even when the lasing area is very large, thereby allowing high-power, single-mode oscillation over a large cavity area at the photonic band edge [12,13].

## 2. Methods

Epitaxial layers were grown using a Vecco Gen-II solid-source MBE. Our MBE is equipped with a RHEED (reflection high-energy electron diffraction) system for *in situ* diagnostics through the monitoring of crystallographic surface features. Conventional effusion cells were used for group III and dopant elements, whereas valved crack cells were adopted for arsenic and antimony. We also opted “epi-ready” n^+^-doped GaSb (001) substrates. In the loading chamber, the substrate was heated to 200 °C to desorb water vapor. In the buffer chamber, the substrate was heated to 300 °C for the desorption of organic residues before being transferred to the growth chamber pressurized to $1\times 10^{-10}$ Torr. Prior to growth, the native oxide was desorbed under surface stabilization. Completed layers underwent rapid cooling without Sb flux to ensure mirror-like surfaces, i.e., without an Sb film. No evidence of surface decomposition was observed.

The process of growing bulk AlGaAsSb was optimized before initiating the growth of a full laser structure. The epitaxial growth procedure began with the desorption of the native oxide layer from the surface of the *n*^+^-doped GaSb substrate in the growth chamber under Sb_2_ stabilization at ~520 °C. We then grew a 200-nm GaSb buffer layer followed by a 500-nm Al_0.3_Ga_0.7_As_0.026_Sb_0.974_ or Al_0.5_Ga_0.5_As_0.043_Sb_0.957_ or Al_0.85_Ga_0.15_As_0.072_Sb_0.928_) layer, and a 20-nm GaSb cap layer. Note that the targeted Al molar fractions were determined by the group-III growth rates. Then, the samples were removed and analyzed via high-resolution X-ray diffraction (HRXRD) using a Bede D1 diffractometer (Jordan Valley, Durham, UK) with Cu $K_{\alpha_{1}}$ radiation (λ≈1.54056 Å) and monochromatized X-ray beam. All of the diffraction spectra presented in this paper are 2θ/ω scans for 004 reflection.

After obtaining the epitaxial growth parameters for Al_x_Ga_1−x_As_y_Sb_1−y_ lattice-matched to GaSb, we grew a strained QW device. This involved the growth of an *n*-GaSb buffer layer followed by a 250-nm waveguide layer of n-Al_0.3_Ga_0.7_As_0.026_Sb_0.974_, and then an undoped active region (for a double-QWs structure). The active region comprised two pairs of 10-nm In_0.35_Ga_0.65_As_0.095_Sb_0.905_ QW and a 20-nm Al_0.3_Ga_0.7_As_0.026_Sb_0.974_ barrier. The barriers were produced using the same nominal concentration as the waveguide layers. Sb_2_ was overpressurized to stabilize the growth of the In_0.35_Ga_0.65_As_0.095_Sb_0.905_ active layer, and a small flux of As_2_ was used to control lattice matching. This resulted in In_0.35_Ga_0.65_As_0.095_Sb_0.905_ QWs under high compressive strain. Finally, the laser structure was completed by applying a *p*-Al_0.3_Ga_0.7_As_0.026_Sb_0.974_ waveguide layer and a 20-nm contact layer of strongly *p*-doped GaSb. During the growth of these layers, As_2_ and Sb_2_ were provided by valved crackers operating at 840 °C and 1050 °C, respectively. The resulting samples were analyzed using HRXRD and atomic force microscopy (AFM, Bruker, Santa Barbara, CA, USA), and optical performance was evaluated using photoluminescence (PL, HORIBA Scientific, NJ, USA) spectroscopy.

**Figure 2:**
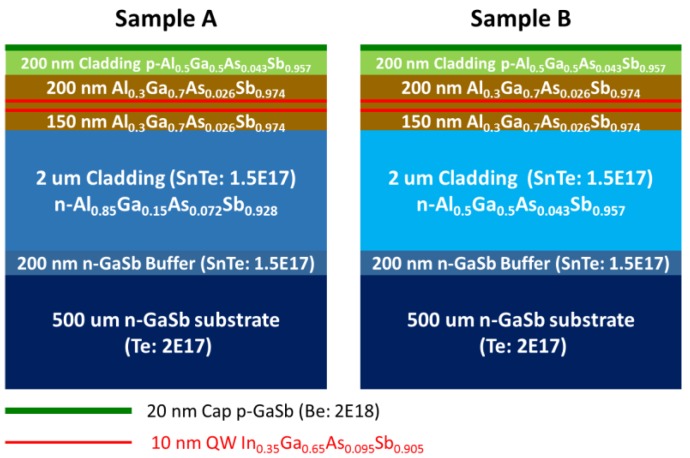
Schematic diagrams of two different type-I InGaAsSb/AlGaAsSb quantum well (QW)/barrier laser structures (samples A and B) for application in photonic crystal surface-emitting laser (PCSELs). The only difference between the two devices was the composition of the *n*-cladding layer.

In this study, we grew two different type-I InGaAsSb/AlGaAsSb QW/barrier laser structures (sample A and B) for application in PCSELs. The fabrication process used for the two devices was the same; however, the composition of the bottom cladding layer differed slightly. We began with a 200-nm *n*-GaSb buffer layer, over which a two-um *n*-Al_0.85_Ga_0.15_As_0.072_Sb_0.928_ cladding layer (sample A) or *n*-Al_0.5_Ga_0.5_As_0.043_Sb_0.957_ cladding layer (sample B) was grown. Over the cladding layer, a 150-nm undoped Al_0.3_Ga_0.7_As_0.026_Sb_0.974_ waveguide layer was grown, followed by an undoped active region (double-QWs structure). The active region comprised two 10-nm In_0.35_Ga_0.65_As_0.095_Sb_0.905_ QWs separated by a 20-nm barrier layer of Al_0.3_Ga_0.7_As_0.026_Sb_0.974_. Above the active region, the laser structure was completed by applying a 200-nm waveguide layer of undoped Al_0.3_Ga_0.7_As_0.026_Sb_0.974_, a cladding layer of *p*-Al_0.5_Ga_0.5_As_0.043_Sb_0.957_, and a 20-nm contact layer of highly *p*-doped GaSb. Figure 2 presents the epitaxial structure of samples A and B in accordance with the design outlined in [14]. After growth, the samples were analyzed using HRXRD, AFM, and PL.

**Figure 3:**
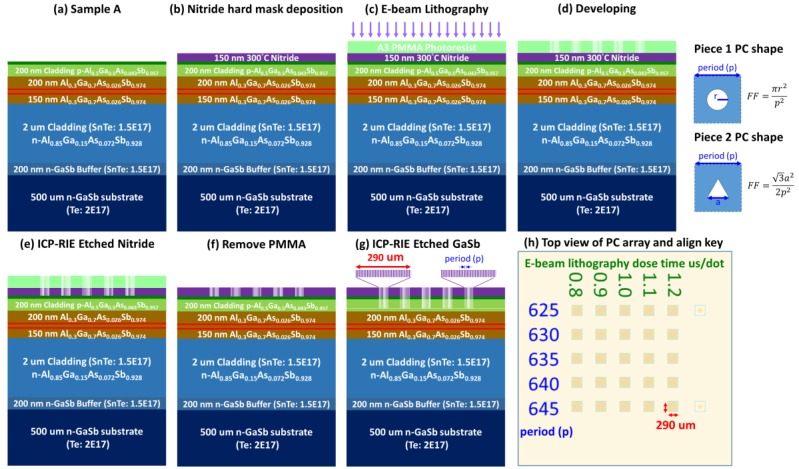
Fabrication process of PCSEL device based on sample A. (**a**) Epitaxy structure; (**b**) deposition of 150 nm Si_3_N_4_; (**c**) defining photonic crystal by EBL; (**d**) developing; (**e**) transferring pattern to Si_3_N_4_ by ICP; (**f**) PMMA removing; (**g**) etching to desired depth by ICP; (**h**) top view of PCSEL sample for optically-pumped measurements.

Samples A and B were cut into several pieces (10 mm × 8 mm). Circular and triangular photonic crystals were respectively created on the first and second pieces of sample A. Figure 3 presents a schematic illustration showing the process flow for an optical pumping PCSEL. The PCSELs were designed for a two-dimensional (2D) square lattice in transverse electric (TE) mode operated at the Γ band-edge. Prior to PC patterning, a 150-nm dielectric layer of Si_3_N_4_ was deposited as a hard mask using plasma-enhanced chemical vapor deposition (PECVD, Oxford Instruments, Bristol, UK) at 300 °C. PCs with circular or triangular-shaped air holes were then defined using e-beam lithography (EBL, ELIONIX INC., Tokyo, Japan)) and dry-etched using inductively coupled plasma (ICP, Oxford Instruments, Bristol, UK)). The ICP etching depth was 200 nm [15].

Based on simulation results, we determined that the air-hole fill factor (FF) of 0.08 (defined as the area of the unit lattice occupied by air holes) would provide the best light coupling, and thereby reduce the threshold gain of the square-lattice PCSEL [16]. Therefore, we set the FF at 0.1. We patterned a series of lattice periods (p) and split the PC parameter of the lattice period between 625–670 nm in steps of five nm to produce a wavelength close to the QW peak gain. This included the fabrication of a 5 × 5 array or a 6 × 6 array of PC regions within each piece. Each PC region covers 290 × 290 μm^2^. The EBL current was set at 50 pA, and the dose time for each dot was from 0.8~1.2 us. We employed a 1064-nm pulsed fiber laser optically pumped using a pulse duration of 50 ns and a repetition rate of five KHz. Measurements were temperature-controlled using a thermoelectric cooler. Light emissions were collected using a monochromator equipped with an InGaAsSb detector. The highest spectral resolution was 0.05 nm.

## 3. Results and Discussion

Table 1 lists the optimized growth parameters (including the V/III ratio, Sb_2_/As_2_ ratio, and group III growth rate) of the target bulk lattice-matched AlGaAsSb alloys at ~480 °C and strained In_0.35_Ga_0.65_As_0.095_Sb_0.905_ at ~440 °C. The group-III growth rate was calibrated against the GaAs substrate. Normalizing the lattice constants of the substrates made it possible to achieve the target alloy composition on the GaSb substrate. Note that Table 1 shows the growth rate before normalization.

**Figure 4:**
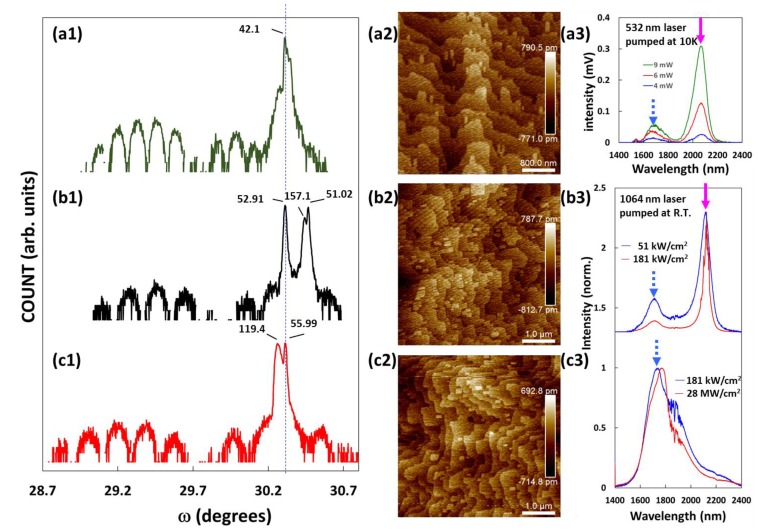
High-resolution X-ray diffraction (HRXRD), atomic force microscopy (AFM), and photoluminescence (PL) results obtained from the (**a**1–**a**3) QW device; (**b**1–**b**3) sample A; and (**c**1–**c**3) sample B. In the rocking curves, the blue dotted line indicates the peak position of the GaSb substrate, whereas the numbers near each peak are the full width at half maximum (unit: aresec). The rocking curves are offset vertically for clarity. The blue dotted arrows and pink arrows in the PL results indicate the emissions of Al_0.3_Ga_0.7_As_0.026_Sb_0.974_ and In_0.35_Ga_0.65_As_0.095_Sb_0.905_, respectively. The input power or power density of the laser are also shown in the PL curves.

Figure 4 presents the HRXRD, AFM, and PL results from the QW device, sample A, and sample B. The QW/barrier periodicity produced a series of satellite peaks. Despite our efforts to ensure the stability of epitaxy in each run, there remained some discrepancies in the position of the HRXRD peaks. In the growth of two group-V components, we found that the Sb_2_/As_2_ ratio affected the peak position of the Al_x_Ga_1−x_As_y_Sb_1−y_ layer in the rocking curves. The growth temperature had little effect on the peak position of the Al_x_Ga_1−x_As_y_Sb_1−y_ layer; however, it did have an effect on the incorporation coefficients of group-V elements and change y. If the active regions were non-relaxed, then the satellite peaks would be clearly resolved. Good control over temperature resulted in sharp interfaces between the QW and barrier layers, as indicated by the narrow satellite peaks of high intensity in the HRXRD results. The rate of arsenic incorporation was proportional to the temperature [17]; therefore, we attribute the discrepancies in peak positions to the influence of the substrate holder on temperature control during the growth process.

The AFM images in Figure 4 show the morphology of the GaSb cap layer. All of the AFM results revealed two-dimensional terraces that had surface roughness comparable to one monolayer (ML) [9]. The bottom cladding layer of sample A was grown at 520 °C, whereas the bottom cladding layer of sample B was grown at 480 °C. Al_x_Ga_1−x_As_y_Sb_1−y_ materials with higher Al content are best grown at elevated temperatures (>500 °C) [18]. Thus, the active region was grown at 440 °C, and subsequent regions were grown at 440 °C to a depth of 10 nm, and then maintained at 480 °C.

The PL spectra revealed peaks associated with In_0.35_Ga_0.65_As_0.095_Sb_0.905_ and Al_0.3_Ga_0.7_As_0.026_Sb_0.974_ in Sample A, but not in Sample B. Using almost the same design as our Sample B, Kaveh et al. [18] grew a ridge waveguide diode laser with emission in the 2.5~2.7 um wavelength range. Our sample B differed from the sample in Kaveh’s study only in terms of growth temperature and the thickness of the top cladding layer. Our sample B presented signs of deterioration in the active region due to the *in situ* annealing during the growth of the upper cladding layer.

**Figure 5:**
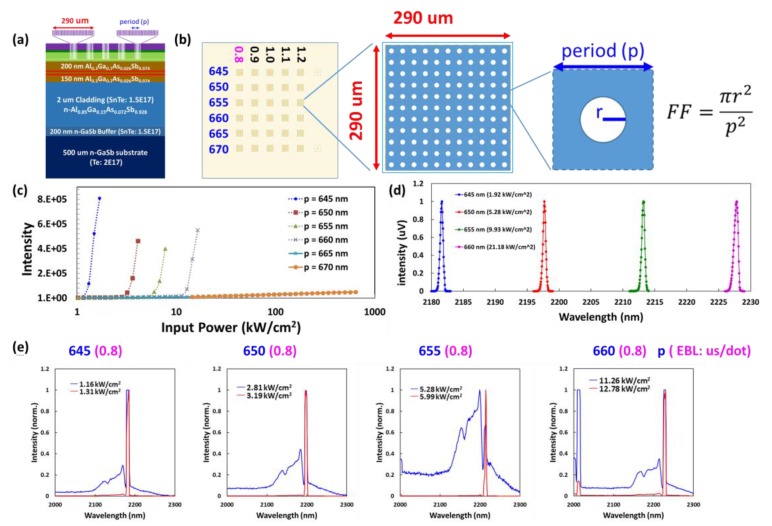
(**a**) Cross-section view and (**b**) top view of sample A; the figures are not drawn to scale (piece 1). (**c**) Light-in versus light-out (L-L) curve of a circular PCSEL device with periodicity between 645–670 nm and an e-beam lithography (EBL) dose duration of 0.8 us/dot. (**d**) Normalized above-threshold lasing spectra and (**e**) normalized emission spectra in the immediate vicinity of the lasing threshold for the lasingable period (645 nm to 660 nm) with an EBL dose duration of 0.8 us/dot. The lasing wavelength was red-shifted approximate 15 nm for each five-nm step in the lattice period between 645–660 nm.

**Figure 6:**
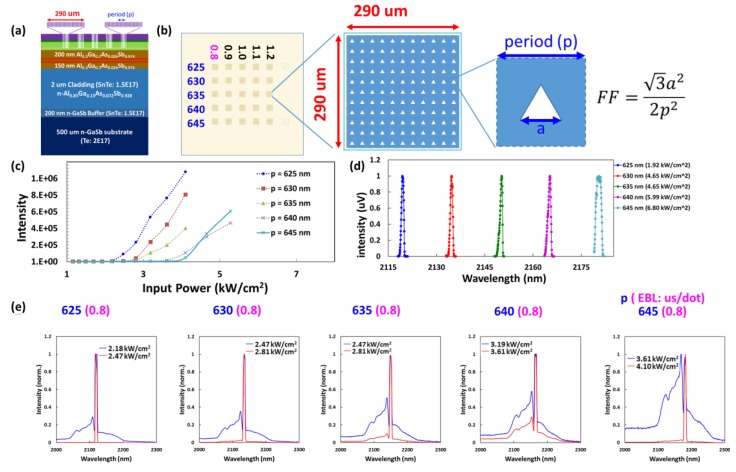
(**a**) Cross-section view and (**b**) top view of sample A; the figures are not drawn to scale (piece 2). (**c**) L-L curve, (**d**) normalized above-threshold lasing spectra, and (**e**) normalized emission spectra in the immediate vicinity of the lasing threshold of a triangular PCSEL device with periodicity between 625–645 nm produced using an EBL dose duration of 0.8 us/dot. The lasing wavelength was red-shifted approximately 15 nm for each five-nm step in the lattice period between 625–645 nm.

The PC parameter of the lattice period was split between 625 nm and 670 nm in steps of five nm to produce a wavelength around the QW gain peak. When the EBL dose duration was changed from 0.8 us/dot to 1.2 us/dot for the same period (645 nm) in the circular PC, the lasing wavelength was almost the same (i.e., approximately 2181 nm for sample A). This indicates that the dose duration had a negligible effect on the lasing wavelength (results not shown). In the optically pumped experiments, no lasing was observed in devices with a period exceeding 665 nm. Figure 5 and Figure 6 present log-log/log-linear plots of light-in versus light-out (L-L) curves and normalized emission spectra in the immediate vicinity of the lasing threshold, as derived from the device in Figure 3. The output characteristics in Figure 5 and Figure 6 are typical of those obtained from any laser: (1) light output changes from a broad spectrum below a particular threshold (blue line) to a narrow spectrum above a threshold (red line); (2) output power presents a nonlinear dependence on input power, with a kink at the lasing threshold (L-L curve) [19]. The spectrum below the threshold reveals two or three band-edge modes on the short-wavelength side in the lasing mode. As for the threshold power density (*P*_th_) and detuning wavelength (Δ*λ*_detuning_), the relationship between PC *λ*_resonant_ and QW gain *λ*_peak_ (~2180 nm) is as follows: a higher detuning wavelength corresponds to a lower QW gain and higher threshold power density.

The lasing wavelength of the PCSEL was close to the peak wavelength in the normal pumping PL spectra from the grown sample. The threshold power of the circular PCSEL devices was significantly lower than the threshold power of the grown samples. When the lattice period was increased from 645 nm to 660 nm, the threshold power increased from 1.16 kW/cm^2^ to 11.26 kW/cm^2^. This can be attributed to the wavelength detuning between the resonant wavelength of the PC and QW peak gain (Figure 5). Thus, increasing the gain for future PCSEL patterns would require decreasing the lattice period while maintaining the same FF.

Increasing the lattice period of the triangular PC from 625 nm to 645 nm led to an increase in the threshold power density from 2.18 kW/cm^2^ to 3.61 kW/cm^2^. This can be attributed to the wavelength detuning between the resonant wavelength of the PC and the QW peak gain (Figure 6). The lasing wavelength of PCSEL is a function of the lattice period, etching depth, and FF. Since the empirical parameter of the effective refractive index is determined by correlating the lasing wavelength with the lattice period (n_eff_ = λ/p), the effect of the depth and FF remains hidden. For a given periodicity, the triangular PC presented higher power and lower threshold power density, compared to the circular PC.

The PCSEL devices that were fabricated using sample B did not present lasing characteristics (results not shown). We did not observe a noticeable kink in the L-L curves or a pronounced narrowing of the linewidth in the normalized emission spectra in the immediate vicinity of the lasing threshold. Sample A differed from sample B only in terms of Al concentration in the bottom cladding layer: sample A (Al_0.85_Ga_0.15_As_0.072_Sb_0.928_) and sample B (Al_0.5_Ga_0.5_As_0.043_Sb_0.957_). Based on the confinement factor and lasing characteristics, sample A is preferred over sample B for PCSEL applications.

Although our device structure is quite similar to that previously reported in reference [14], we use this further for electrically pumped PCSEL fabrication. The key technique [20] by controlling the Group-V elements at interfaces is also opted to enhanced the optical properties in quaternary GaInAsSb/AlGaAsSb quantum wells in this manuscript. However, our previous work [14,20] only stopped at optically pumped PCSEL performance. The performance difference between this manuscript and our previous work is owing to the different conditions of our MBE system. In our recent work, we further measured the photonic band structure of a square lattice with a circular unit cell for photonic crystal. The measurement apparatus for the photonic band structure is comprehensively described in the Supplementary Materials (Figure S1,S2). We really saw the grating effect of photonic crystal, no matter whether it was after optically pumped or electrically pumped fabrication processes (the complete fabrication process diagram is also shown in the Supplementary Materials (Figure S3)). However, some issues during the fabrication process (such as annealing, metallization, Indium Tin Oxide (ITO) deposition, and nitride deposition) will affect the surface lasing phenomena in electrically pumped devices. We will continuously study the effects of these issues on lasing performance to reach successful electrically pumped devices.

## 4. Conclusions

This paper describes the influence of growth conditions on an InGaAsSb/AlGaAsSb QW/barrier GaSb-based type-I laser structure. We measured the V/III ratio, Sb_2_/As_2_ ratio, and group-III growth rate of target bulk lattice-matched AlGaAsSb alloys and strained In_0.35_Ga_0.65_As_0.095_Sb_0.905_. By incorporating the band-edge modes of square-lattice PC in QW laser epitaxy, we observed surface lasing only for sample A. Samples A and B differed only in the composition of the bottom cladding layer: Al_0.85_Ga_0.15_As_0.072_Sb_0.928_ (sample A) and Al_0.5_Ga_0.5_As_0.043_Sb_0.957_ (sample B). For a given periodicity, the triangular PC presented higher power and lower threshold power density, compared to the circular PC.

Sample B did not present surface lasing phenomena due to the deterioration of the active region during the growth of the upper cladding. The growth temperature was shown to have a negligible effect on the HRXRD peaks in the Al_x_Ga_1−x_As_y_Sb_1−y_; however, determining the optical properties of laser devices is a critical issue. Higher Al content in the bottom cladding layer and lower Al content in the top cladding layer is suggested for PCSEL applications.

## Figures and Tables

**Table 1:** Optimized growth parameters of target bulk AlGaAsSb alloys at ~480 °C and strained In_0.35_Ga_0.65_As_0.095_Sb_0.905_ at ~440 °C.

Parameter	Sb_2_/A_2_	V/III	r_Al_ (um/hr)	r_Ga_ (um/hr)	r_In_ (um/hr)
Al_0.30_Ga_0.70_As_0.026_Sb_0.974_	~11.9	~8.57	0.175	0.4	
Al_0.50_Ga_0.50_As_0.043_Sb_0.957_	~6.4	~7.19	0.4	0.4	
Al_0.85_Ga_0.15_As_0.072_Sb_0.928_	~6.2	~8.57	0.684	0.12	
In_0.35_Ga_0.65_As_0.095_Sb_0.905_	~3.8	~3.45		0.4	0.264

## Supplementary Materials

The following are available online at http://www.mdpi.com/1996-1944/12/2/317/s1, Figure S1: The measurement apparatus for photonic band structure; Figure S2: The measured photonic band structure (dotted lines) in (**a**) the *Γ*-(+X) and *Γ*-(-X) direction and (**b**) the *Γ*-(+M) and *Γ*-(-M) direction; Figure S3: Fabrication process of electrically pumped PCSEL device based on sample A.
