# Supplementary material for: PCSEL Performance of Type-I InGaAsSb Double-QWs Laser Structure Prepared by MBE
Source: Materials (Basel). 2019 Jan 21;12(2):317. doi: 10.3390/ma12020317 (PMC6357140; doi:10.3390/ma12020317)
Supplement: Supplementary file 1 [file materials-12-00317-s001.pdf]

materials

Supplementary Materials

PCSEL Performance of Type-I InGaAsSb Double-QW Laser Structure Prepared by MBE

Hui-Wen Cheng [1,2,*], Shen-Chieh Lin [2], Zong-Lin Li [2], Kien-Wen Sun [2,3] and Chien-Ping Lee [2]

[1] Center for Nano Science and Technology, National Chiao Tung University
[2] Department of Electronics Engineering and Institute of Electronics, National Chiao Tung University
[3] Department of Applied Chemistry, National Chiao Tung University
* Correspondence: hwcheng2011@gmail.com; Tel.: +886-3-5712121-ext55632

1. Photonic band structure of photonic crystal

The measurement apparatus for the photonic band structure is shown in Figure S1: (i) A 1064 laser passed through one reflection mirror and focused by a lens is incident on the sample on the Cu stage. (ii) Emission from the sample surface was collected into an optical fiber. The fiber was placed on a manual-controlled rotating arm. (iii) The spectrum was measured using an InGaAsSb multichannel spectrometer having a resolution of approximately one nm. (iv) By rotating the arm around the sample, a series of angle-dependent spectra were measured for polar angles from 0 to ±10°, in steps of 2°. The sample was manually rotated on the Cu stage in order to change the in-plane direction from $\Gamma - X$ to $\Gamma - M$.

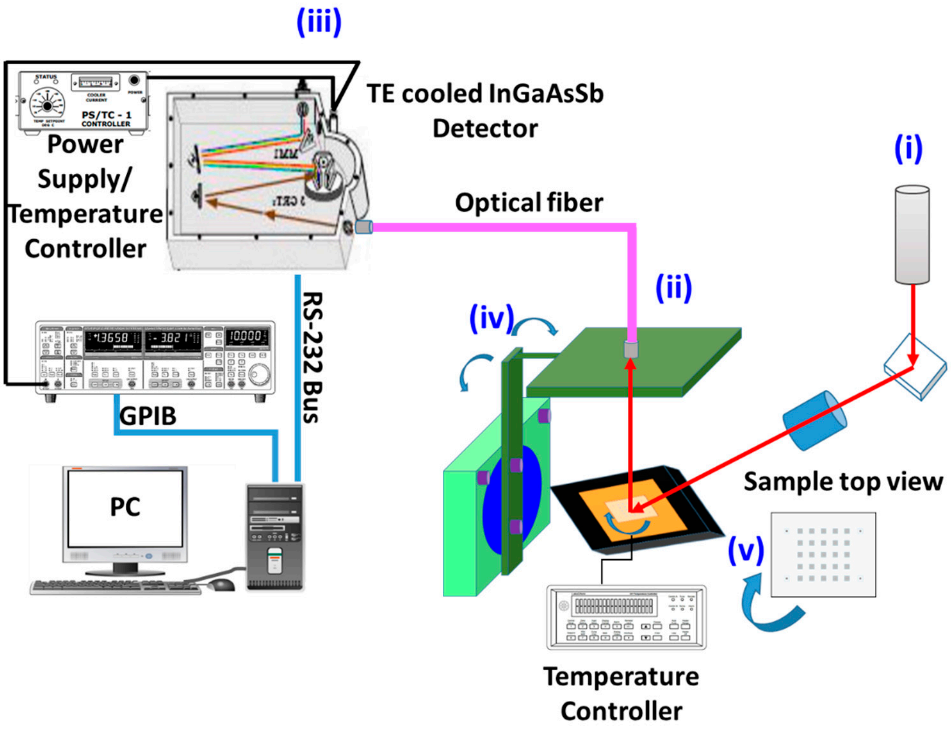

Figure S1. The measurement apparatus for photonic band structure.

The band structure was measured by observing the resonance coupling of light from an active layer to the bands of the photonic crystal. The output light from the device is coupled to an optical fiber and then transferred to a monochromator with an InGaAsSb detector. Below the threshold current, the emission spectrum was broad, and four peaks were observed with a distribution similar to the photonic band structure. By contrast, the spectrum became sharp with a peak width of ~2170 nm above the threshold (taking period 630 as example), which was equal to the resolution limit of the measurement system. For a given frequency, resonance coupling occurs when the

in-plane wavevector of light matches ($k_{\parallel}$) the wavevector of the photonic bands ($k_p \sim \frac{2\pi}{a_{\Gamma-X}}$). In spontaneous emission spectra, this coupling can be observed as a sharp peak. The in-plane wavevector is related to the polar angle θ by $k_{\parallel} \sim \frac{2\pi}{\lambda} \cdot \sin\theta$, where λ is the wavelength in free space. By changing the polar angle θ around a direction normal to the plane and shifting the in-plane direction from $\Gamma - X$ to $\Gamma - M$, we were able to map out the photonic bands around the Γ-point. The normal direction corresponds to the Γ-point.

The measurement results are shown in Figure S2. These results are similar to those measured by Noda [1,2]. Noda's spectrometer and rotating arm were computer-controlled so that the angle-dependent spectra could be automatically measured. Besides, their sample was mounted on a rotating stage in order to change the in-plane direction from $\Gamma - X$ to $\Gamma - M$. Therefore, their results had higher resolution and a smoother band spectrum.

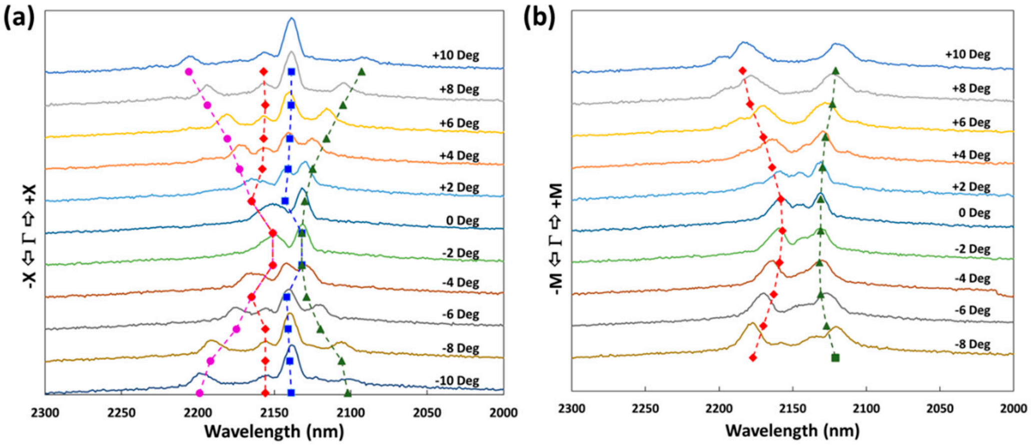

Figure S2. The measured photonic band structure (dotted lines) in (a) the Γ-(+X) and Γ-(-X) direction and (b) the Γ-(+M) and Γ-(-M) direction.

2. Fabrication Process of Electrically Pumped PCSEL Device

After the further fabrication of our sample for electrically pumped devices (Figure S3), we still measured a similar photonic band structure, as shown in Figure S2 (the data was not shown). This means that the grating effect of photonic crystal still works after these complicated fabrication processes. However, the device didn't have surface lasing phenomena (i.e., electrically pumping failed). Yet, the same sample went through only processes (a) to (h) in Figure S3; it really exhibited surface lasing phenomena (i.e., optically pumping was successful). Some issues occurred between (i) and (y) in Figure S3 that need to be overcome in order to fabricate successful electrically pumped devices. We are now preparing another manuscript to discuss all of these issues, including the effects of annealing, metallization, ITO deposition, nitride deposition… etc. on the surface lasing phenomena in electrically pumped devices.

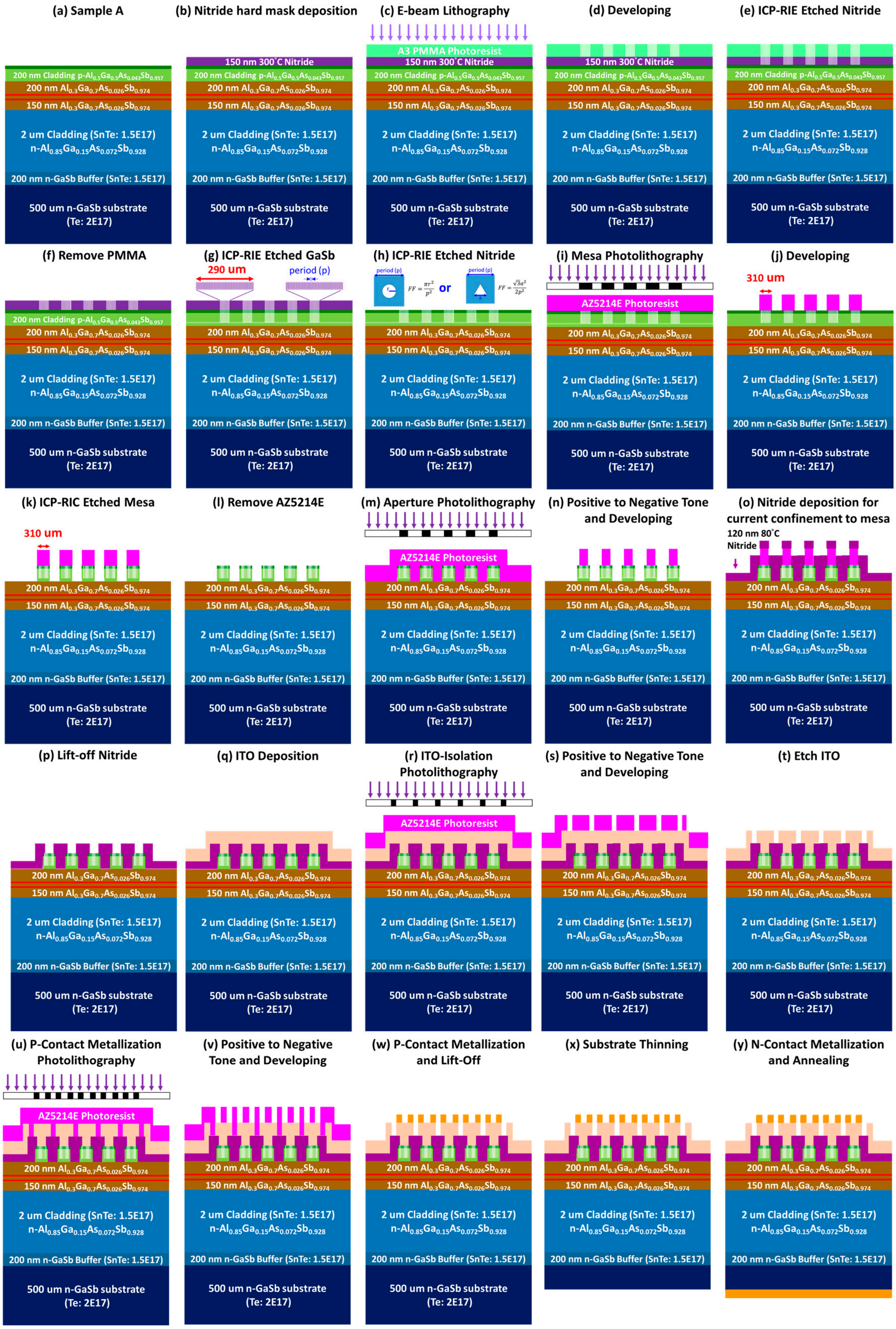

Figure S3. Fabrication process of electrically pumped PCSEL device based on sample A.

References

1. Pan, C. H.; Lin, C. H.; Chang, T. Y.; Lu, T. C.; Lee, C. P. GaSb-based mid infrared photonic crystal surface emitting lasers. *Opt. Express* **2015**, 23, pp 11741-11747.

2. Lin, C. H.; Lee, C. P. Enhanced optical property in quaternary GaInAsSb/AlGaAsSb quantum wells. *Solid State Commun.* **2014**, 116, pp 153504.

© 2018 by the authors. Submitted for possible open access publication under the terms and conditions of the Creative Commons Attribution (CC BY) license (http://creativecommons.org/licenses/by/4.0/).
